# Extracellular Hemoglobin, Hypoxia, and Macrophage-Mediated Pulmonary Vascular Remodeling in Hemolytic Disease

**DOI:** 10.3390/ijms27188170

**Published:** 2026-09-14

**Authors:** Melissa J. Lucero, Eva Nozik, Kathryn Hassell, David C. Irwin, Paul W. Buehler, Scott K. Ferguson

**Affiliations:** 1 Translational Research Laboratory of Red Blood Cell Diseases and Hypoxia Related Illnesses, Division of Cardiology, School of Medicine, University of Colorado Anschutz Medical Campus, Aurora, CO 80045, USA; melissa.lucero@cuanschutz.edu (M.J.L.);; 2 Cardiovascular Pulmonary Research Group, School of Medicine, University of Colorado Anschutz Medical Campus, Aurora, CO 80045, USA; eva.nozik@cuanschutz.edu; 3 Pediatric Critical Care Medicine, Department of Pediatrics, University of Colorado Anschutz Medical Campus, Aurora, CO 80045, USA; 4 Division of Hematology Colorado Sickle Cell Treatment and Research Center, School of Medicine, University of Colorado Anschutz Medical Campus, Aurora, CO 80045, USA; 5 Center for Blood Oxygen Transport and Hemostasis, School of Medicine, University of Maryland, Baltimore, MD 21201, USA; 6 Integrative Aerospace and Exercise Physiology Laboratory, Embry-Riddle Aeronautical University, Daytona Beach, FL 32114, USA

**Keywords:** sickle cell disease, pulmonary hypertension, macrophages, iron, hemoglobin, heme, exercise tolerance, haptoglobin, and hemopexin

## Abstract

Pulmonary hypertension (PH) is a well-recognized complication of chronic hemolytic anemias such as sickle cell disease and thalassemia, yet the relative contributions of hypoxia and cell-free hemoglobin (Hb) to disease progression remain incompletely understood. Patients with hemolytic anemia experience a lifelong cycle of chronic and inter bitten hypoxia that compounds vascular injury driven by extracellular Hb and its degradation products, heme and iron. While the effects of hypoxia and Hb exposure have historically been studied in isolation, the combined impact of sustained, low-level plasma Hb together with chronic hypoxia—more representative of steady-state hemolysis—has been largely overlooked. A rat model incorporating chronic hypoxia with continuous low-dose Hb infusion via an implanted pump demonstrates that even modest plasma Hb concentrations (10–20 µM heme) exert an additive effect on hypoxia-induced PH. This effect is associated with increased adventitial macrophage accumulation, oxidative stress, and inflammation, driving more severe pulmonary vascular remodeling. Building on this model, therapeutic strategies targeting Hb-mediated vascular injury are evaluated, with particular focus on repeated-dose haptoglobin (Hp) therapy, given that Hp is often severely depleted in sickle cell disease. Restoring circulating Hp sequesters plasma Hb into a non-reactive, compartmentalized Hb–Hp complex, limiting NO scavenging and oxidative damage. These mechanistic findings are further linked to functional outcomes through studies of skeletal muscle microvascular oxygen tension and exercise capacity in Berkeley sickle cell disease mice. This review synthesizes findings across these studies to clarify the interplay between hypoxia, macrophage biology, and extracellular Hb in driving pulmonary vascular remodeling and to highlight emerging Hb-targeted therapeutic strategies for hemolysis-associated PH.

## 1. Introduction

Pulmonary hypertension (PH) as a sequela of hemolytic anemias [1,2,3] occurs at a higher frequency when compared to the incidence of primary PH in the general population [4,5,6,7]. The current definition of PH is dependent on a pulmonary arterial catheter measurement of >20 mmHg at rest [8,9], which replaces the former > 25 mmHg value, reflecting evidence that mildly elevated pressures carry adverse prognostic significance [8]. PH associated with hemolytic diseases is variable with different distinct phenotypes [8]. This review focuses on the vasculopathy associated with pre-capillary PH. Despite substantial mechanistic work on acute, high-concentration Hb exposure and NO scavenging, the pathophysiological consequences of chronic, low-level plasma Hb exposure (more representative of the steady-state hemolysis seen in conditions such as sickle cell disease and thalassemia) remain poorly defined, particularly in combination with hypoxia that these patients experience over a lifetime. Prior studies have largely examined hypoxia or Hb exposure in isolation, leaving the additive or synergistic effects of these two clinically relevant “hits” on pulmonary vascular remodeling insufficiently characterized. Moreover, while perivascular/adventitial macrophage accumulation has been implicated in hypoxia-driven PH, its specific relationship to extracellular Hb exposure and its contribution to an “outside-in” model of pulmonary vascular remodeling in the hemolytic setting has not been systematically reviewed. Finally, despite growing interest in Hb scavenging as a therapeutic strategy, there is a lack of consolidated evidence connecting haptoglobin-based therapies to functional, whole-body outcomes such as exercise capacity in hemolytic disease.

This review addresses these gaps by synthesizing pre-clinical studies specifically designed to isolate and combine continuous low-dose Hb exposure with chronic hypoxia. Thus, allowing for direct evaluation of their additive effects on pulmonary hemodynamics, vascular remodeling, and adventitial macrophage phenotype. We define the objective and scope of this review as fourfold: (1) to consolidate mechanistic evidence on how chronic hypoxia and continuous extracellular Hb exposure interact to drive perivascular macrophage accumulation and pulmonary vascular remodeling; (2) to evaluate haptoglobin-based and other Hb-targeted therapeutic strategies tested in this model as potential interventions for hemolysis-associated PH; (3) to extend these mechanistic findings to translational, whole-body measures of microvascular oxygenation and exercise intolerance in a humanized mouse model of sickle cell disease; and (4) connect these findings to current clinical practice. Together, this review aims to bridge a critical gap between biochemical Hb/NO/heme biology and its downstream cardiopulmonary and functional consequences in chronic hemolytic anemia.

**Search strategy and selection criteria:** The literature on sickle cell and beta thalassemia–associated pulmonary hypertension was reviewed through June 2026. Literature searches were conducted on PubMed using a combination of terms including: hemoglobin, nitric oxide, hypoxia, macrophage, hemolytic diseases, sickle cell disease, beta thalassemia, hemolytic diseases, haptoglobin, hemopexin, ferroportin, critical speed, pulmonary hypertension, pulmonary vascular remodeling, and right ventricular hypertrophy. Terms were combined for a systematic PubMed search, including (not limited to) hemoglobin AND nitric oxide, hemoglobin AND hypoxia, hemoglobin AND pulmonary hypertension, sickle cell disease AND pulmonary hypertension, and beta thalassemia AND pulmonary hypertension. Results were screened for relevance to pulmonary hypertension related to hemoglobin/hemolysis and hypoxia and to protein-based therapies related to haptoglobin, hemopexin, and ferroportin inhibition.

Pulmonary vascular remodeling caused by hemolytic anemia disease states has multifactorial mechanisms that ultimately lead to remodeling [9]. The most well-studied and accepted mechanism of chronic extracellular Hb-induced PH is the abnormal regulation of nitric oxide (NO) signaling [10,11]. NO depletion is reported to be associated with the generation of reactive oxygen species such as superoxide (O_2_•^−^) resulting from increased circulating or vascular wall associated xanthine oxidase [12,13]. Under these pro-oxidant conditions, nitric oxide (NO) rapidly reacts with superoxide (O_2_•^−^) to form peroxynitrite (OONO^−^), a toxic reactive nitrogen species [14]. Peroxynitrite is a key mediator of nitrosative/oxidative damage, promoting nitration and oxidation of proteins, disruption of lipid membranes, and impairment of heme iron chemistry [14]. By both consuming NO to further reduce its bioavailability and generating additional damaging oxidants, the NO–O_2_•–peroxynitrite pathway can reinforce endothelial dysfunction and vascular remodeling in hemolytic anemias [14]. Evidence of higher Hb oxidation states (ferric-Fe^3+^ and ferryl-Fe^4+^) as well as tissue globin chain crosslinking has also been reported following exposure to Hb [15,16,17,18,19].

Hb can consume NO via two reactions: (I) NO dioxygenation of oxygenated Hb (HbFe^2+^O_2_), which generates nitrate anion (NO_3_^−^) and ferric Hb (HbFe^3+^), and (II) iron nitrosylation of deoxyHb (HbFe^2+^), a reaction that occurs by direct iron binding of NO to deoxygenated ferrous Hb in the ^2+^ redox state.

(I) Hb-Fe^2+^(O_2_) + NO• → [Hb(Fe^3+^OONO•)] → Hb-Fe^3+^ + NO_3_^−^
(II) Hb-Fe^2+^ + NO• → Hb(NO)

Both reactions result in acute and chronic vasoactivity depending on the extent and duration of exposure to extracellular Hb. Hemolysis-induced systemic and pulmonary hypertension can contribute to vascular remodeling in response to a state of NO depletion [20].

Increased tissue concentrations of peroxides [i.e., hydrogen peroxide (H_2_O_2_) or lipid peroxides] occur in several diseases, particularly within the context of inflammation during cycles of hypoxia/ischemia and reperfusion that occur in PH associated with hemolytic anemia. Biochemical reactions of Hb with physiological pro-oxidants (peroxides) have been studied in vitro and to some extent in vivo [21,22]. Reaction of Hb with peroxide (H_2_O_2_) can be summarized as follows: deoxy-Hb (HbFe^2+^) reacts with H_2_O_2_ generating (III) oxo-ferryl Hb [Hb(Fe^4+^=O)], [21] ferric Hb [Hb(Fe^3+^)], and (V) protein radical formation [•Hb(Fe^4+^=O)] [23].

(III) Hb(Fe^2+^) + H_2_O_2_ → Hb(Fe^4+^=O) + H_2_O
(IV) Hb(Fe^4+^=O) + H^+^ → Hb(Fe^3+^) OH^−^
(V) Hb(Fe^3+^) + H_2_O_2_ → •Hb(Fe^4+^=O) + H_2_O

Globin chain free radicals that are produced during reaction (III) can oxidize amino acid and lipids within the Hb protein or free radicals can transfer to molecules such as lipoproteins, creating lipid–protein adducts (4-hydroxy-nonenal, 4-HNE). During free radical reactions within Hb, a defined peptide region that is located at the α-globin/β-globin interface is the primary site of amino acid oxidation. In particular, the β-chain Cys93 as well as the α-chain Tyr42 are the primary amino acids affected following ferryl Hb radical [•Hb(Fe^4+^=O)] formation, leading to protein unfolding, intermolecular crosslinking, and progressive degradation of the Hb molecule into precipitated protein, heme, and iron [24,25,26].

## 2. Heme, Hypoxia, and Macrophages

In hemolytic anemia-associated PH, chronic hypoxia may amplify hemolysis-driven vascular oxidative stress by promoting mitochondrial reactive oxygen species production, impairing redox homeostasis, and contributing to nitric oxide depletion and endothelial dysfunction. To investigate this mechanism, our group evaluated the effects of extracellular Hb on pulmonary hemodynamics and remodeling using a reductionist approach. In this study, programmable pumps were implanted into Sprague Dawley rats to deliver cell-free Hb at low (12 mg/day; 0.5 mg/h) and high (35 mg/day; 1.45 mg/h) doses over 3, 5, and 7 weeks under normoxic and hypoxic (10% O_2_, 5500 m; 18,000 feet; barometric pressure 380 mmHg) conditions [21]. In this model, the Hb infusion concentration is in the range of 3–10 μM heme and consistent with values of SCD patients [5,19,21]. Under normoxic conditions, significant Hb-induced elevations in pulmonary arterial pressures were observed, and this effect was further amplified under hypoxic conditions. These findings support an additive relationship between chronic hypoxia and continuous, low-level extracellular Hb exposure in driving pulmonary vascular remodeling, and were associated with increased adventitial macrophage accumulation, oxidative stress, and inflammation within the pulmonary vasculature.

Clinical studies investigating pre-capillary PH in the SCD population also observe both hypoxia and cell-free Hb mechanisms driving the disease processes [1]. Observations show downstream effects of hemolytic anemia, including hypercoagulability, inflammation, and tissue hypoxia, which are likely coupled with individual genetic susceptibility, resulting in altered expression of a wide panel of genes [27]. Notable pathways associated with vascular remodeling, including Wnt signaling, calcium signaling, vascular smooth muscle contraction, and cancer, were represented in patients with elevated right systolic pressures, suggesting that these pathways are likely to play a contributory role in the development of PH [27].

Strong evidence also suggest that a hypoxic and Hb-rich microenvironment leads to a pro-oxidative and inflammatory macrophage phenotype [28]. Several groups have evaluated the role of inflammation on the initiation and progression of PH in hypoxia, scleroderma, parasitic infections and anemia [29]. Stenmark et al. have compelling results suggesting that fibroblasts and immunomodulatory cells including macrophages and other cell types contribute to the expansion of the pulmonary vascular vaso vasorum during hypoxia [30,31,32]. This occurs because of the activation of cells residing within the adventitia of vessels and leads to chronic generation of reactive oxygen species [33] and inflammation, causing an “outside-in” induction/ progression of pulmonary vascular remodeling and PH [31,33,34].

## 3. Haptoglobin

A critical role for tissue-resident monocytes/macrophages is the clearance of Hb-haptoglobin (Hp) complexes. Hp is the primary Hb-binding protein in plasma and tissue of mammals and serves to buffer increases in cell-free hemoglobin during hemolysis. In humans, Hp can exist as one of three primary phenotypes (Hp 1-1, Hp 2-1, and Hp 2-2) in a range of concentrations from 0.3–1.9 mg/mL [35]. All three Hp polymorphisms share the same Hb-binding β globin (Hpβ) but differ in α globin chains (Hpα1 or Hpα2), giving rise to dimeric (Hp 1-1) or multimeric (Hp 2-1, Hp 2-2) structures, depending on the number of α globin cysteine residues involved in disulfide bond formation [35]. In humans, Hb-Hp complexes are cleared from circulation by the scavenger receptor CD163 expressed on the surface of tissue resident monocytes/macrophages [35,36]. CD163 expression is restricted to cells on the monocyte-macrophage lineage with high cell surface expression in Kupffer cells of the spleen and liver [37]. Upon binding of Hb-Hp to CD163, the complex is internalized by endocytosis to the endosomal-lysosomal compartment, where heme is degraded by heme oxygenase-1 (HO-1) ultimately to biliverdin with the release of carbon monoxide (CO) [37]. Iron is released and bound to its cellular storage protein ferritin, and CD163 is recycled back to the cell surface [37].

**Figure 1:**
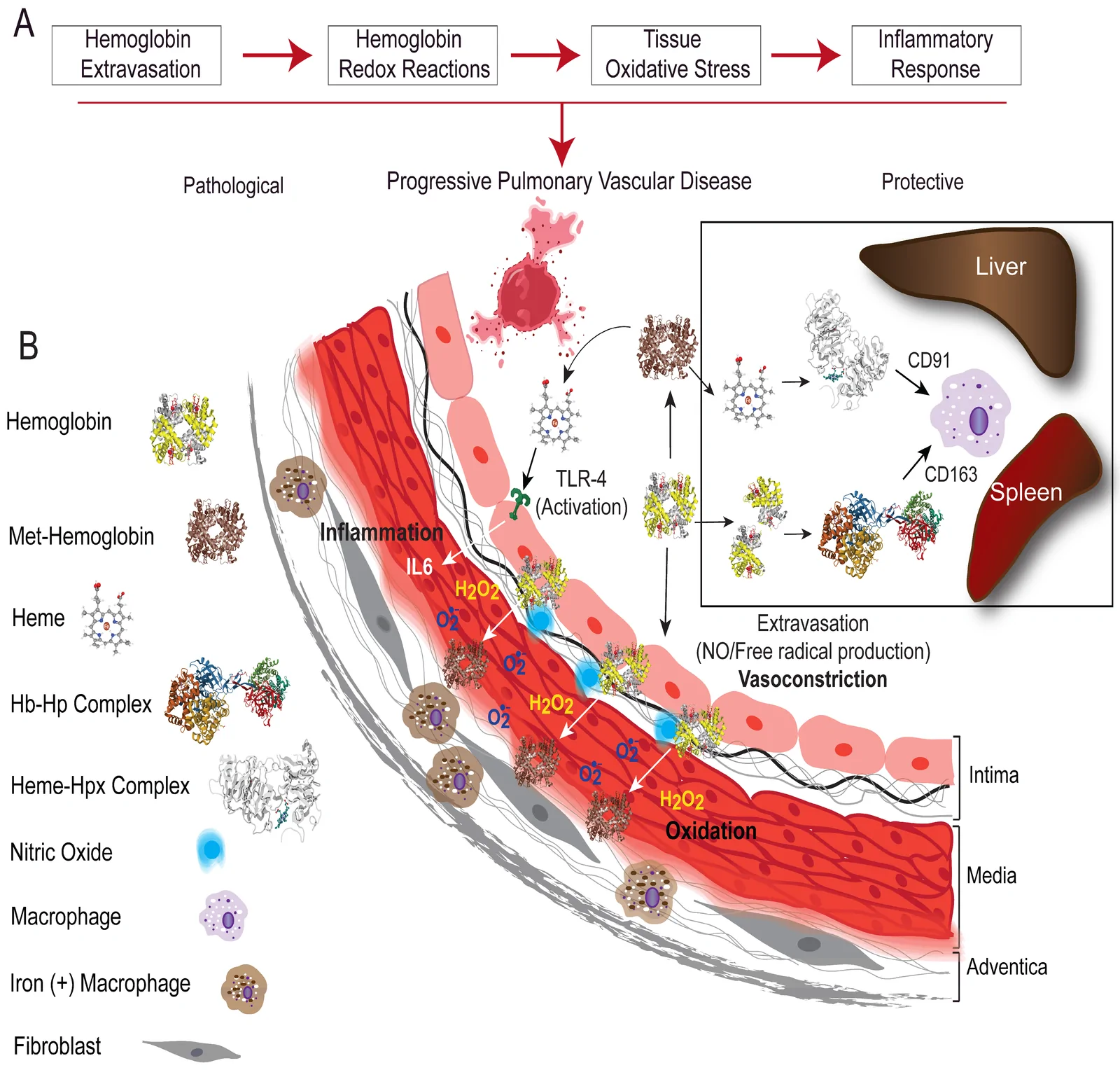
(**A**) The sequential progression from Hb access into the perivascular space (extravasation) to factors that lead to the progression of pulmonary vascular disease are described. (**B**) Describes the pathological role of Hb following extravasation into the perivascular space. In this environment Hb can oxidize to met-Hb and readily releases heme and iron. Both can contribute to tissue lipid peroxidation that leads to reactive lipid adduction to enzymatic and non-enzymatic proteins. This tissue injury can lead to the recruitment of macrophages to the adventitia and differentiation of fibroblasts within this compartment. All of these factors lead to inflammation and the progression of PH. However, increasing the Hp pool prevents the induction of the Hb driven cascade of events by driving the process toward Hp binding, vascular compartmentalization, and clearance by liver and spleen tissue-resident macrophages that express high CD163 and HO-1 (CD163^+^/HO-1^+^).The haptoglobin-Hb structure was generated from PDB ID: 4F40 [39]; the hemopexin-heme structure was generated from PDB ID: 1QHU [40].

Heme also drives changes in macrophage phenotype polarization. A heme-driven macrophage phenotype with high HO-1 expression, high antioxidant capacity, and suppressed HLA class 2 has been consistently found in atherosclerotic plaques with intra-plaque hemorrhage [37]. Schaer and colleagues demonstrated that Hb complex formation with Hp leads to macrophage polarization favoring a CD163—HO-1—HLA class 2^low^—antioxidative phenotype, which is alternatively activated to support healing at the site of injured tissue. This scenario likely differs in our model in that Hb extravasation, oxidation, heme, and iron release leads to tissue injury followed by inflammatory cell recruitment and accelerated remodeling. However, expansion of the circulating Hp pool through repeated administrations could intercept this cascade upstream, preventing the initial event of extravasation event and limiting Hb contribution to tissue injury. Supporting this concept, twice-weekly Hp administration (90 mg/kg, i.v.) in a Hb plus hypoxia rat model (Hb 35 mg/day; 1.45 mg/h and hypoxia-10% O_2_, 5500 m; 18,000 feet; barometric pressure 380 mmHg) mitigated the Hb effects [38] (Figure 1).

Experimental observations from this study confirmed that extracellular Hb gains access to perivascular spaces in the pulmonary vasculature, leading to a cascade of Hb oxidation and accumulation of reactive non-heme iron within the vascular tissue. This created a tissue microenvironment favorable for oxidation/inflammation, as visualized by increased 4-HNE immunoreactivity, accumulation of inflammatory cells (including CD163+ macrophage populations) within the vascular adventitia, and a significant increase in inflammatory molecules ICAM-1 and IL-6 [38,41]. Moreover, iron accumulation was observed in cardiomyocytes and around the cardiac blood vessels of the right heart. Together these findings suggest that the progression of Hb- and hypoxia-induced pathophysiology led to enhanced metrics of cardiopulmonary remodeling.

## 4. Hb Extravasation, NO Insensitivity, and Macrophage-Dependent Remodeling

Beyond its role in amplifying hypoxic vascular remodeling, it is well recognized that Hb either in the vessel lumen and/or its extravasation into perivascular compartments drives vascular dysfunction [1,2]. Insights gained from a variety of preclinical models demonstrates that perivascular Hb accumulation causes insensitivity to NO [1], vasoconstriction, inflammation, and endothelial and smooth muscle cell dysfunction [1]. In addition, like other forms of PH, recent evidence now supports a critical role for lung recruitment of circulating macrophages to contribute to PH in hemolytic disease [34,42]. However, data cannot definitively resolve the temporal sequence between intravascular NO scavenging by luminal free Hb and the subsequent adventitial iron/macrophage accumulation, as these processes likely occur on overlapping timescales rather than as discrete, sequential steps. It is plausible that intraluminal NO consumption alone—via endothelial dysfunction and downstream signaling to the adventitia—contributes to early fibroblast activation prior to, or independent of, detectable Hb extravasation.

Evidence for a distinct macrophage phenotype driving pulmonary vascular remodeling comes from lung tissue of deceased SCD-PH patients [34], where severe vascular remodeling—intimal thickening, plexiform lesions, medial hypertrophy—colocalized with iron deposits and CD163^+^ cells expressing HO-1, ET-1, and IL-6. This pattern points to a macrophage phenotype shaped by chronic hypoxia and Hb exposure. An important knowledge gap remains for iron accumulation in the macrophages, and the mechanism for recruitment to the pulmonary adventitia. Iron accumulation in the macrophage is likely driven predominately by extra vascular hemolysis and erythrophagocytosis. An emerging hypothesis is that complement factor C3b binds to phosphatidyl serine and CR1on damaged sickled red cells targeting them for macrophage clearance [2,43]. Although, in humans the clearance of cell free Hb via the haptoglobin and CD163 receptor on macrophages is also recognized to contribute to iron loading. Macrophage recruitment to the pulmonary adventitia is also currently an area of investigation. Like other types of PH including hypoxia and schistosomiasis, the adventitial fibroblast likely plays a critical role in this process by upregulating IL-6 and its complement [33,44,45,46]. In SCD-PH and other forms of PH, it is still not clear where the root source of recruited macrophages originates, whether from the circulating monocyte pool or resident macrophages in the spleen or liver. Macrophage depletion via GdCl3 in Hb plus hypoxia-exposed rats lowers pulmonary artery pressures, reduces RV hypertrophy, and attenuates vascular remodeling; However, GdCl_3_ is toxic to circulating macrophages as well as liver and spleen macrophages, making the assessment of any specific macrophage pool as a root source contributing to disease progression difficult [34,47].

The macrophage phenotype also appears to evolve over time. Early in Hb plus hypoxia exposure (day 18), perivascular macrophages express ET-1; by day 35, iron-laden macrophages additionally express HO-1 and IL-6. This combination—clearance-associated HO-1 alongside vasoactive and inflammatory mediators—suggests a disease-specific phenotype distinct from a purely protective scavenger role. Macrophage depletion, which reduces iron accumulation and remodeling, further supports macrophages as a mechanistic link between hemolysis, iron deposition, hypoxia, and disease progression.

Whether macrophages remain uniformly injurious or shift function during disease resolution has also been examined [48]. Following Hb plus hypoxia exposure and subsequent recovery under normoxic, Hb-free conditions, untreated animals show partial regression of PH: pulmonary pressures fall, RV function improves, and remodeling lessens. Macrophage depletion during this recovery period impairs resolution, resulting in higher pressures and more remodeling than in non-depleted animals [47]. A shift from CD163 to CD68 expression in depleted lungs suggests altered macrophage subpopulations rather than complete elimination, which may implicate distinct macrophage phenotypes in both injury and repair [34,48]. Nevertheless, interpreting these recovery kinetics requires caution given the broad, off-target systemic effects of unchelated lanthanides. Because GdCl_3_ simultaneously blocks L-type calcium channels and alters tissue iron handling [47], these alternative, non-immune mechanisms must be considered before attributing functional changes solely to macrophage elimination.

Molecular changes during recovery reinforce this dual role. IL-6 declines regardless of macrophage depletion, indicating that removal of Hb and hypoxia alone is sufficient to reduce this inflammatory signal. ET-1, in contrast, remains elevated during recovery unless macrophages are depleted, identifying macrophages as a persistent source of vasoactive signaling. HO-1 expression rises in macrophage-depleted lungs, particularly in vascular cells, suggesting compensatory stress responses that do not fully resolve underlying injury. Collectively, these findings support a model in which macrophages function as harmful effectors during active hemolysis-driven PH, while also participating in vascular recovery—with phenotype and timing determining whether their net effect promotes remodeling or resolution.

## 5. Therapeutic Targeting of Hb, Heme, and Iron Handling

**Figure 2:**
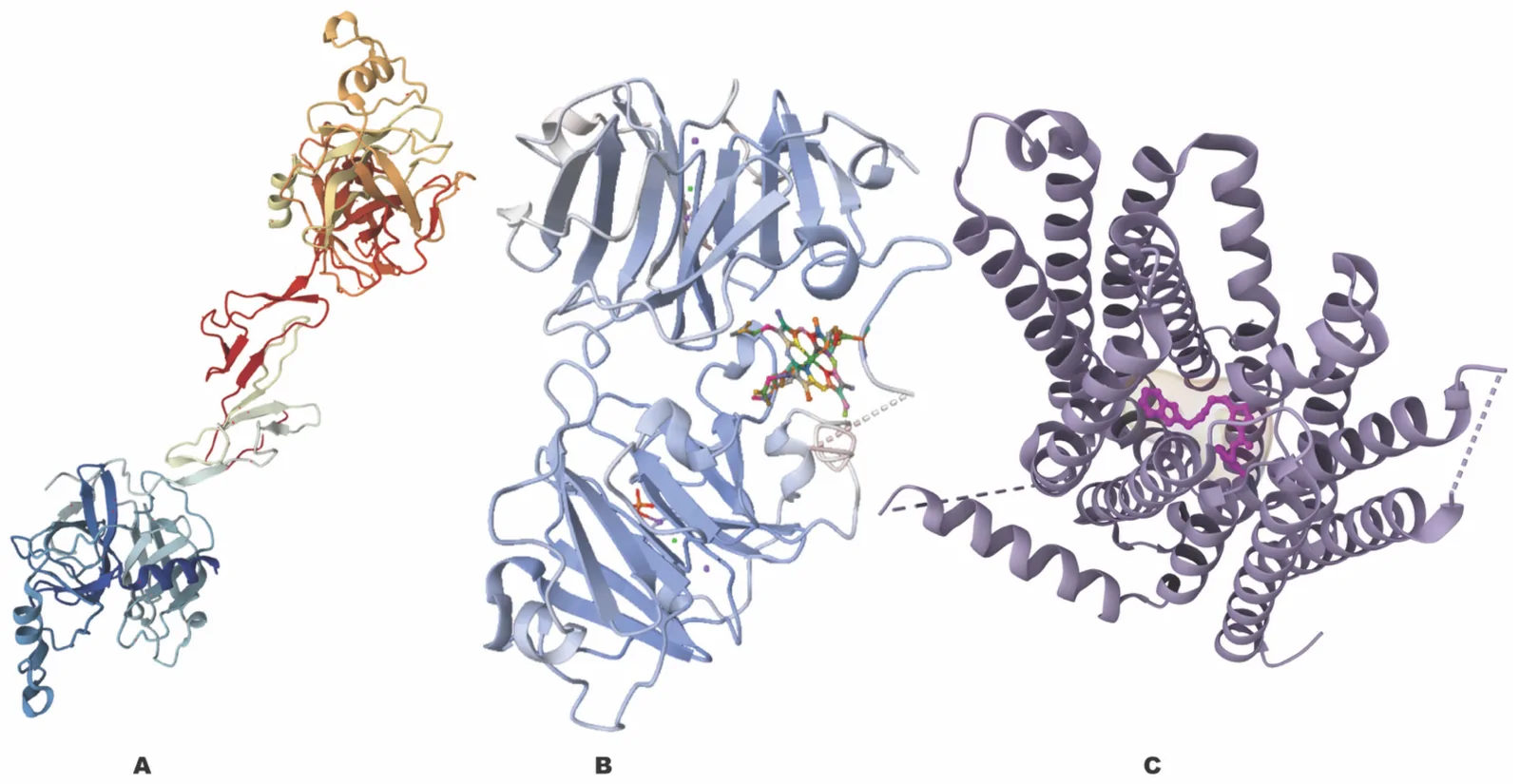
**Therapeutic molecules for iron scavenging or restriction.** (**A**) One of the forms of haptoglobin (Hp), (**B**) hemopexin (Hpx) with bound heme, and (**C**) SLC40/ferroportin in complex with vamifeport in an outward facing conformation.

Chronic hemolysis promotes oxidative injury to plasma lipoproteins once the primary Hb and heme scavengers, Hp and hemopexin (Hpx), become depleted [35] (Figure 2A,B), consistent with prior evidence that lipoproteins participate in plasma heme consumption and lipid oxidation. In SCD patient plasma, elevated free Hb, total heme, and non-transferrin-bound iron accompanied Hp exhaustion and reduced Hpx, suggesting that heme iron increasingly transfers to lipoproteins as scavenger capacity is lost—positioning iron as an active driver, not merely a byproduct, of vascular injury [49]. Berk-SS mice recapitulated this pattern, showing greater lipid oxidation than wild-type mice following heme-albumin exposure [41]. Post-mortem lung tissue from SCD-PH patients further linked this oxidative process to macrophage-rich vascular pathology, with oxidized LDL deposition in thickened intima/adventitia and strong uptake by perivascular macrophages—implicating macrophages as amplifiers of hemolysis-driven oxidative injury and remodeling.

## 6. Peripheral Oxygen Transport and Critical Speed as a Functional Endpoint

Beyond its vascular effects, Hb-mediated NO scavenging also impairs peripheral oxygen transport: acute cell-free Hb exposure reduces contracting skeletal muscle microvascular PO_2_ to a degree similar to NOS inhibition, an effect largely prevented when Hb is complexed with Hp [50]. This peripheral mechanism, combined with pulmonary vascular dysfunction, contributes to exercise intolerance in SCD. Supporting this, Berk-SS mice housed at mild-to-moderate altitude for three months showed exacerbated hemolysis, elevated RV systolic pressure, disrupted eNOS/endothelin balance, and reduced exercise capacity relative to sea-level controls [51], demonstrating that even modest hypoxia worsens cardiopulmonary and functional outcomes in SCD.

**Figure 3:**
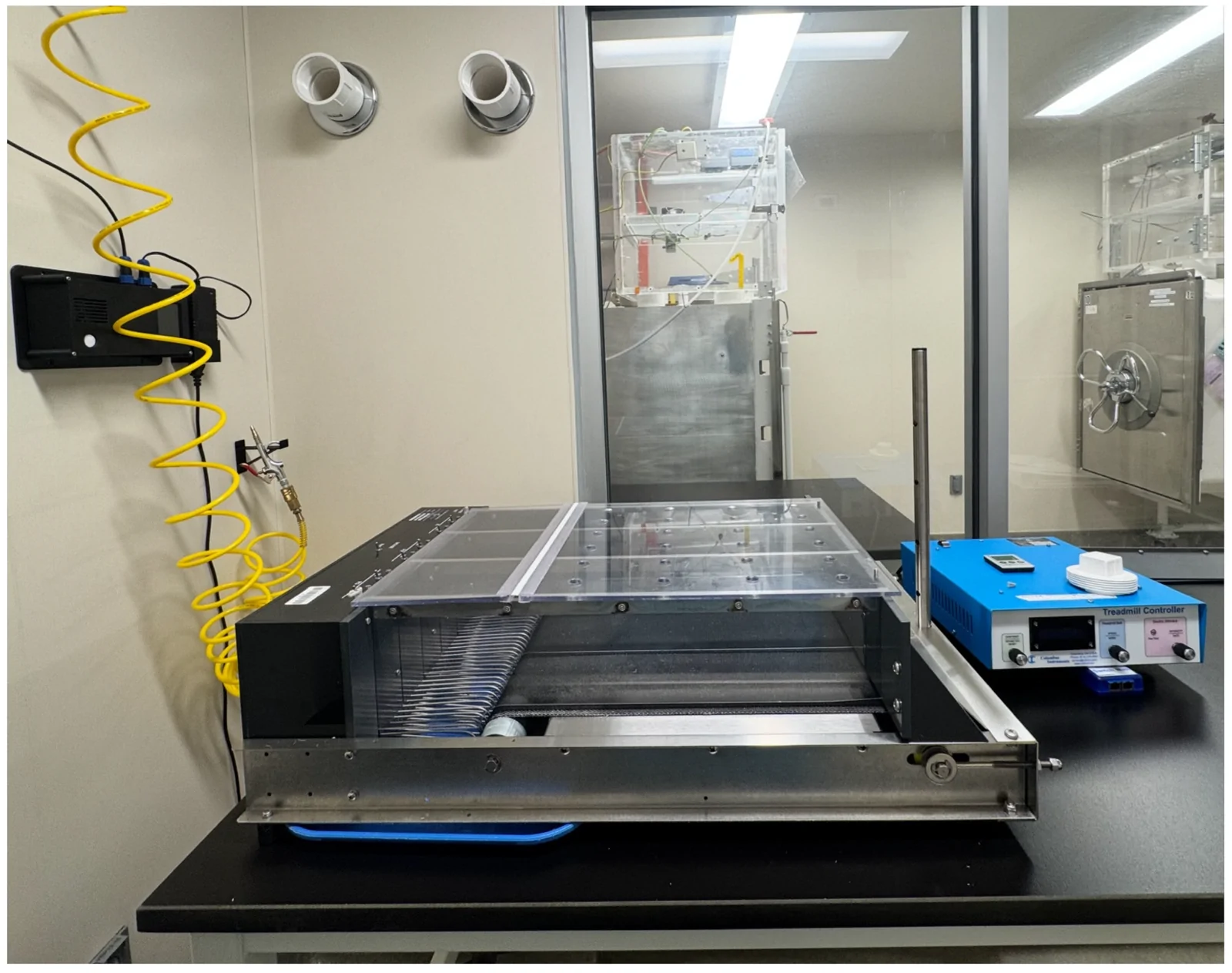
**Experimental design for critical speed.** The treadmill has isolated rows where the mice can run (with air burst motivation) for critical speed measurement at various angles and speeds. This treadmill resides in a room where critical speed can be measured under normoxic or hypoxic conditions.

Critical speed (CS)—the maximal sustainable exercise speed that can be supported by aerobic metabolism [50,51,52,53,54,55]—has emerged as a preclinical functional endpoint analogous to the six-minute walk test in humans (Figure 3), integrating central cardiopulmonary function with peripheral O_2_ delivery-utilization matching [50,51,52,53,54,55]. Dietary nitrate supplementation, which normalized CS in Berk-SS mice [52], along with the PO_2_mv findings above, established the mechanistic rationale for using CS to evaluate heme-scavenging therapies.

This framework was applied to test hemopexin supplementation in hypoxia-stressed Berk-SS mice with progressive cardiopulmonary disease, motivated by human plasma data showing Hpx depletion, heme excess, and lipoprotein oxidation in SCD, alongside autopsy evidence of severe pulmonary vascular remodeling and RV damage. Berk-SS mice exposed to three months of hypobaric hypoxia and treated with low- or high-dose subcutaneous Hpx showed dose-dependent improvement in CS, with high-dose Hpx significantly exceeding vehicle controls [38]. This benefit occurred without correcting anemia (unchanged hematocrit and spleen weight), indicating that Hpx acts by mitigating heme toxicity rather than restoring the red cell mass. Hpx also improved RV mechanics—reduced stiffness, increased stroke volume and cardiac output, lower afterload, and better ventricular-vascular coupling—though RV systolic pressure and hypertrophy showed only partial reversal. Histologic and multi-omic analyses corroborated these functional gains: Hpx treatment reduced iron-laden macrophages, lipid peroxidation, fibrosis, and vascular remodeling in the lung, and attenuated oxidative injury and interstitial fibrosis in the RV [38]. Imaging confirmed interstitial extravasation of both Hb and heme, supporting a model in which Hpx neutralizes tissue heme toxicity to improve both cardiopulmonary function and exercise performance [38].

## 7. Heme/Hb Scavenging: Local Delivery and Combination Therapy

Extending systemic Hpx replacement, subsequent work explored more targeted heme-scavenging strategies. Because iron and heme accumulate preferentially in pulmonary adventitial and perivascular regions, aerosolized Hpx delivery was tested as a means of concentrating heme neutralization in the lung while limiting systemic exposure [56]. Twice-weekly intrapulmonary Hpx administration for 10 weeks localized to alveolar and perivascular regions without substantial systemic uptake, reduced pulmonary iron deposition and oxidative markers (4-HNE, HO-1), and improved exercise capacity (~16% increase in critical speed) independent of anemia correction [56]. RV stiffness, afterload, stroke volume, cardiac output, and ventricular-vascular coupling all improved, alongside reduced pulmonary vascular resistance and a trend toward less medial thickening. Proteomic profiling supported these functional gains, showing restoration of antioxidant (thioredoxin, glutathione) and iron-handling pathways in the lung and reduced remodeling/inflammatory signaling in the RV.

A related strategy combined Hp and Hpx to simultaneously scavenge Hb and free heme, both of which are depleted in SCD [57]. In Berk-SS mice exposed to chronic hypoxia to accelerate PH development, twice-weekly Hp + Hpx therapy produced a similar physiologic and molecular profile: improved RV mechanics (lower stiffness and Ea, higher cardiac output, better ventricular-vascular coupling), reduced pulmonary vascular resistance and medial hypertrophy, and proteomic/metabolomic shifts consistent with decreased oxidative stress, inflammation, and fibrotic signaling in both lung and RV [57]. Notably, both approaches converged on a common cellular finding: iron-laden perivascular macrophages, present in human SCD-PH lung tissue and recapitulated in Berk-SS mice, were reduced following treatment. This suggests that limiting extracellular Hb/heme burden—whether through pulmonary-targeted or combined systemic scavenging—reduces macrophage iron loading and its downstream contribution to oxidative injury and vascular remodeling.

## 8. Iron Restriction via Ferroportin Inhibition

Rather than intercepting Hb or heme extracellularly, an alternative strategy targets iron handling within macrophages and erythroid cells after Hb/heme uptake. Ferroportin inhibitors limit cellular iron export and have shown efficacy in several disease models [58,59,60,61,62]; in SCD, reduced iron availability can lower mean corpuscular hemoglobin and sickling propensity, potentially attenuating downstream vascular injury.

In Berk-SS mice exposed to subchronic hypoxia, the ferroportin inhibitor VIT-2763 (vamifeport) induced an iron-restricted erythroid phenotype—lower mean corpuscular hemoglobin and volume, hypochromic/microcytic red cells, and fewer sickled cells—without worsening total hemoglobin [62]. This was accompanied by markers of reduced hemolysis: less splenic red cell congestion, lower plasma and renal iron, increased plasma Hpx, and fewer iron-laden splenic macrophages. Cardiopulmonary benefits paralleled these hematologic changes, including reduced RV hypertrophy, lower Fulton index, less pulmonary arterial medial thickening, decreased pulmonary vascular resistance, and improved RV mechanics, shifting treated mice toward a phenotype resembling healthy controls. Proteomic analysis in lung and RV showed changes consistent with improved iron-sulfur cluster homeostasis, reduced oxidative stress, and attenuated inflammatory and remodeling signaling. While this study demonstrates that iron restriction has an overall benefit in attenuating the progression of PH in a murine model, there is a critical knowledge gap in understanding the effects on iron overloaded macrophages. Whether or not, these macrophages become inert iron containing vesicles or become reactive with consequences with immune system function remains unknown.

As in the scavenger-based approaches, iron-laden pulmonary vascular macrophages emerged as a key pathologic feature in both human SCD-PH tissue and Berk-SS mice, and their reduction following VIT-2763 treatment—paralleled by fewer iron-rich splenic macrophages—points to decreased erythrophagocytic iron burden. Collectively, these findings across scavenging and iron-restriction strategies (summarized in Table 1) reinforce that macrophages are active participants in iron handling, oxidative stress, and vascular remodeling in SCD-PH, rather than passive markers of hemolysis.

**Current clinical practice:** Sickle cell disease-associated PH affects approximately 10% of patients and presents as multiple endotypes that include, pre-capillary PAH (5%), CTEPH (2%), and post-capillary PH (3%), with all carrying substantial mortality risk [11]. Clinical guidelines recommend hydroxyurea as first line-line treatment, and advise against PDE5 inhibitors like sildenafil [2]. This is in contrast to guidelines for Beta Thalassemia PH patients that advises use of PDE5 inhibitors as well as other PH therapies including endothelin receptor antagonist, prostaglandin agonist [3]. Also, conditionally support chronic transfusion is advised for both sickle cell disease and beta thalassemia patients [2,3].

**Mechanistic insights for therapeutic development:** From the studies discussed, novel therapeutic development should embrace several principals. First, biomarkers should guide therapy selection. These markers should include the common markers of hemolysis (plasma hemoglobin, haptoglobin, hemopexin, LDH, and arginase) [2] as well as mechanisms associated with inflammation (IL-6; CRP), and iron loading of macrophages (docking of C3b on red blood cells that targets them for erthrrophagocytosis), which may provide an individualized therapy selection [2]. Finally, evidence from preclinical success of NO scavenging and Hp/ Hpx proteins suggests this as a priority [2]. Soluble guanylate cyclase has proven superior to PDE-5 inhibition [8]. Inhibiting arginase also shows efficacy, demonstrating that both substrate availability (L-arginine) and enzyme inhibition merit clinical investigation [2]. Haptoglobin and hemopexin protein-based therapies alongside fetal hemoglobin inducers and ferroportin inhibitors also highlight targeting the root causes. A critical gap in treatment for hemolytic disease-associated PH is lack of any human trials for these developing therapies [2].

## 9. Future Directions

Future studies should define the macrophage phenotypes that emerge when chronic hypoxia, extracellular Hb, heme, lipid oxidation, and iron retention converge in the pulmonary vascular adventitia. Transcriptomic analyses of lung and perivascular macrophage populations after Hb + hypoxia, aerosolized Hpx, combined Hp + Hpx, or iron-restriction therapy could determine whether these cells adopt mixed programs of Hb/heme clearance, iron retention, lipid handling, vasoactive signaling, and inflammatory remodeling. These studies could also test whether hypoxia-induced metabolic reprogramming, including CtBP1-mediated suppression of HO-1 described by the Stenmark group, contributes to a high-iron, low-HO-1 macrophage state that favors remodeling over resolution [63].

Apo/lipoprotein-focused studies would further test whether Hb- and heme-driven lipid oxidation creates macrophage phenotypes that link hemolysis to adventitial inflammation and vascular remodeling. This is relevant because increased intramacrophage iron retention can function as a pro-inflammatory signal [64]. Together, RNAseq and Apo/lipoprotein studies could help distinguish protective scavenger macrophages from iron- and lipid-loaded macrophage populations that actively drive SCD-PH progression.

## 10. Conclusions

Taken together, the studies reviewed herein support a model in which hypoxia amplifies the vascular toxicity of extracellular Hb in hemolytic disorders. Once Hb escapes the vascular lumen and enters perivascular compartments, it consumes NO, undergoes oxidation, releases heme and iron, and promotes lipid peroxidation, inflammation, and vascular remodeling [21,38]. These events are closely associated with perivascular macrophage accumulation, especially iron-laden macrophages within the pulmonary adventitia. In preclinical studies, the skeletal muscle PO_2_mv and CS studies show that beyond the pulmonary vasculature extracellular Hb/NO biology also impairs peripheral O_2_ delivery-utilization matching and whole-body exercise performance. These observations identify several promising therapeutic strategies targeting the upstream components of hemolysis including haptoglobin and hemopexin. In addition, reducing iron release with ferroportin inhibition offers a complementary approach by restricting iron availability, reducing RBC sickling and hemolysis, and limiting accumulation of hyperferric macrophages. Finally, this review highlights the concept that hypoxia, extracellular Hb, heme/iron trafficking, skeletal muscle O_2_ transport, and macrophage biology are tightly linked drivers of hemolysis-associated PH and exercise intolerance.

## Figures and Tables

**Table 1:** Summary of rodent models of Hb/heme toxicity, oxygen transport, and treatments.

Species	Treatment	Dose (per Day)	Route	Altitude	Duration	Refs
**Rat**	Hb	12 mg/d (0.5 mg/h) 35 mg/d (1.45 mg/h)	Infusion pump	18 K	3, 5, 7 weeks	[21] (2012)
	Hb + HX + Hp	Hb 35 mg/d Hp 90 mg/kg	Infusion pump i.v.	18 K	2× weekly	[36] (2015)
	Hb + HbO2 Hb + HbO2-Hp Acetylcholine	35 mg Hb 35 mg Hb Infused over 5 min	Infusion, i.v.	Normoxia	Acute	[37] (2016)
	Cell-free Hb +/− Hp; contracting skeletal muscle PO2mv	Hb 50 mg/kg +/− Hp 50 mg/kg	i.v. bolus	Normoxia	Acute 180-s contractions	[50] (2018)
	Hb + GdCl3 (hypoxic)	Hb 35 mg/d GdCl_3_ 10 mg/kg	iPRECIO SQ pump GdCl3 3x per week	18K	35 days	[34] (2019)
	Hb + GdCl3 (hypoxic recovery)	Hb 35 mg/d GdCl_3_ 10 mg/kg	iPRECIO SQ pump GdCl3 3x per week	18K	5 weeks 4 wk recovery	[48] (2021)
**Guinea pig, mouse**	Hb-Alb	0, 5, 25, 50, 100 µM 5 mg/kg or 25 mg/kg	i.v. infusion	N/A	8 h	[49] (2018)
**Mouse**	Hypoxia with CS test	NA	NA	Sea level 1609 m 2438 m	11 ± 1	[51] (2019)
	Berk-SS + dietary nitrate	1 mmol/kg/day nitrate-rich beetroot juice	Oral supplementation	Sea level	5 days	[52] (2020)
	Hpx	100 or 300 mg/kg	Subcutaneous	8 K	11 ± 1	[38] (2021)
	Hpx	100 mg/kg	Aerosol	8 K	11 ± 1	[56] (2025)
	Hp + Hpx	150 mg/kg each	Subcutaneous	8 K	11 ± 1	[57] (2025)
	VIT-2763	240 mg/kg	Oral gavage	8 K	11 ± 1	[62] (2026)

## Data Availability

No new data were created or analyzed in this study. Data sharing is not applicable to this article.

## Abbreviations

**Abbreviations**		**Hypoxia Equivalencies**
Hb	Hemoglobin	Sea level = 760 mmHg 20.9% O_2_
HX	Hypoxia	5280 ft = 632 mmHg = 17.4% O_2_
GdCl_3_	Gadolinium chloride	8000 ft = 564 mmHg = 15.4% O_2_
Hp	Haptoglobin	18,000 ft = 380 mmHg = 10% O_2_
Hpx	Hemopexin	
Alb	Albumin	
